# Primary Lung Adenocarcinoma Presenting as Cardiac Tamponade in a 40-Year-Old Non-Smoker

**DOI:** 10.7759/cureus.21631

**Published:** 2022-01-26

**Authors:** Noah Dessalegn, Kelsee Felux, Ekram Seid, Amir Mohammed

**Affiliations:** 1 Internal Medicine, Wellstar Atlanta Medical Center, Atlanta, USA; 2 Internal Medicine, Ross University School of Medicine, Miramar, USA

**Keywords:** electrical alternans, metastatic non-small cell lung cancer, malignant pericardial effusion, cardiac tamponade, non-small cell lung adenocarcinoma

## Abstract

Lung cancer is the number one cause of cancer-death in the world with the majority of cases directly attributable to smoking. The diagnosis is mostly made following evaluation for either an incidental lung nodule or respiratory signs and symptoms such as cough and hemoptysis. This is a review of a young never-smoker who presented predominantly with gastrointestinal symptoms, which is an uncommon initial presentation of lung cancer associated with malignant pericardial effusion.

A 40-year-old male without a history of smoking presented with epigastric pain associated with nausea and vomiting. He denied significant cardio-respiratory or systemic symptoms. Physical examination was unremarkable besides tachycardia of 111 beats per minute, blood pressure of 108/65 mmHg, and mild generalized direct abdominal tenderness. EKG showed electrical alternans. CXR demonstrated a prominent cardiac silhouette leading to evaluation with echocardiography, which revealed a large pericardial effusion and signs of cardiac tamponade. 1200 ml of serosanguinous fluid was removed by pericardiocentesis with significant clinical improvement. The basic workup of infectious and immunologic causes was negative, which prompted a contrasted CT scan of the chest. This revealed a left upper lobe mass measuring 3.6 x 2.8 cm without mediastinal or hilar lymphadenopathy. CT-guided biopsy was performed and was consistent with pulmonary adenocarcinoma but was negative for molecular drivers and programmed cell death ligand 1 (PD-L1). Pericardial fluid cytology also confirmed the presence of malignant cells. The patient complained of mild dyspnea and chest pain before discharge which led to a repeat echocardiogram and identification of a recurrent large pericardial effusion. Cardiothoracic surgery consultation was obtained, and the patient underwent subxiphoid pericardial window placement.

Learning points from this case report include: First, non-smoking-related lung cancer is still among the top ten causes of cancer death in the US. It should remain in the differential diagnosis of patients presenting with pertinent signs and symptoms, even in non-smokers. Secondly, malignancy, most importantly primary lung cancer, is a common cause of a large symptomatic pericardial effusion in patients who have a non-revealing basic workup. In such patients, a detailed evaluation for undetected underlying malignancy is important. Thirdly, colchicine and non-steroidal anti-inflammatory drugs are commonly used for the treatment of painful malignant pericardial effusion; however, there is a lack of data to support this practice. Finally, pre-discharge screening echocardiography in patients with new or recurring cardiorespiratory symptoms following initial pericardiocentesis could be important because recurrent large pericardial effusion is a common and potentially fatal complication of malignant pericardial effusion.

## Introduction

With 2.2 million new cases and 1.8 million deaths in the year 2020, lung cancer remains the number-one cause of cancer death in the world [1]. It is estimated that there will be over 130,000 lung cancer deaths in the United States in 2021. Out of this, over 80% are related directly to smoking [2]. Primary cancer of the lung is generally divided into non-small cell lung cancer (NSCLC), comprising over 80% of lung cancers, and small cell lung cancer (SCLC). The diagnosis of lung cancer is mostly made following evaluation for respiratory signs and symptoms. The most common symptoms include cough, hemoptysis, dyspnea, and chest pain. Sometimes, an incidental finding of a nodule or mass during routine imaging can also lead to its diagnosis [3]. We present a case of a 40-year-old Black male with no smoking history who presented with predominantly gastrointestinal symptoms and was found to have large pericardial effusion, the evaluation of which led to a diagnosis of primary adenocarcinoma of the lung.

## Case presentation

A 40-year-old Black male with no pertinent medical history presented with sharp epigastric pain rated an 8/10 onset one week ago associated with nausea and decreased appetite. He reported generalized weakness and mild left-sided chest discomfort that worsened with inspiration and was not associated with exertion. Systemic symptoms of fever and weight loss were denied. He denied cardiovascular symptoms of orthopnea, paroxysmal nocturnal dyspnea, palpitations, and leg swelling. He denied respiratory symptoms including runny rose, sore throat, sneezing, and cough. He denied gastrointestinal symptoms of vomiting and bowel habit changes. He had no known personal history or family history of heart disease. He denied a history of cigarette smoking and drug use other than occasionally smoking marijuana. The patient denied chest and abdominal trauma and recent long-distance travel. When the symptoms started a week ago, he went to an emergency room and was told that he had “fluid in his body.” He was given ibuprofen with instructions to follow up with his primary care physician (PCP). After his symptoms worsened, he went to see his PCP who sent him to our emergency department for further evaluation of these symptoms.

**Figure 1:**
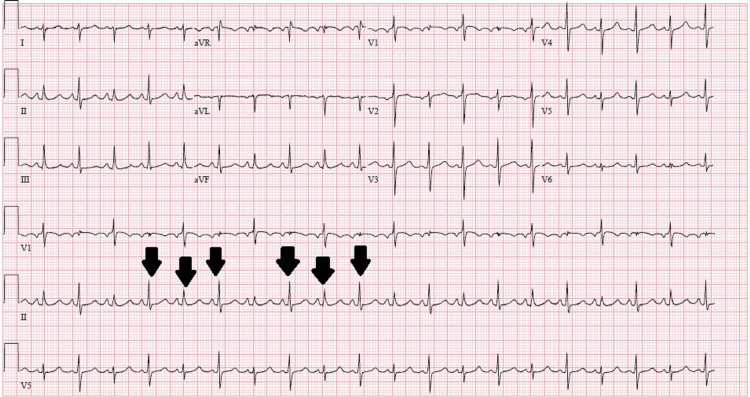
EKG showing electrical alternans (black arrows)

On physical examination, the patient was tachycardiac to 111 bpm, blood pressure was 108/65 mmHg, and oxygen saturation was over 95% on room air. He appeared to be anxious with no signs of labored breathing. He had distended jugular veins, distant heart sounds with no murmurs to auscultation, and no peripheral edema. The chest was resonant to percussion and clear with normal symmetric air entry to auscultation. On abdominal examination, there was mild generalized direct abdominal tenderness without guarding or rigidity and no signs of organomegaly. Laboratory evaluation was significant for hemoglobin of 13 g/dL (14-18 g/dL) and hematocrit of 40% (41-53%). The liver enzymes showed the AST to 89 IU/L (13-39 IU/L) and ALT to 150 IU/L (7-52 IU/L) with elevated bilirubin of 1.8mg/dL (0.0-1.0 mg/dL). Electrolytes, kidney function, and hepatitis markers were unremarkable. Troponin was normal at <0.03 ng/ml (0.00-0.04 ng/ml) and trended over time with no elevation. The brain natriuretic peptide was normal at 74 pg/ml (0-100 pg/ml). Electrocardiography (EKG) showed electrical alternans (Figure 1) and chest X-ray revealed prominent cardiac silhouette and clear lung fields. Subsequently, echocardiography (ECHO) was obtained which showed normal left and right ventricular systolic functions with an ejection fraction of 61-65%, normal valvular structures, a large circumferential pericardial effusion with right ventricular compression, and right atrial inversion which is a sign of cardiac tamponade. 

**Figure 2:**
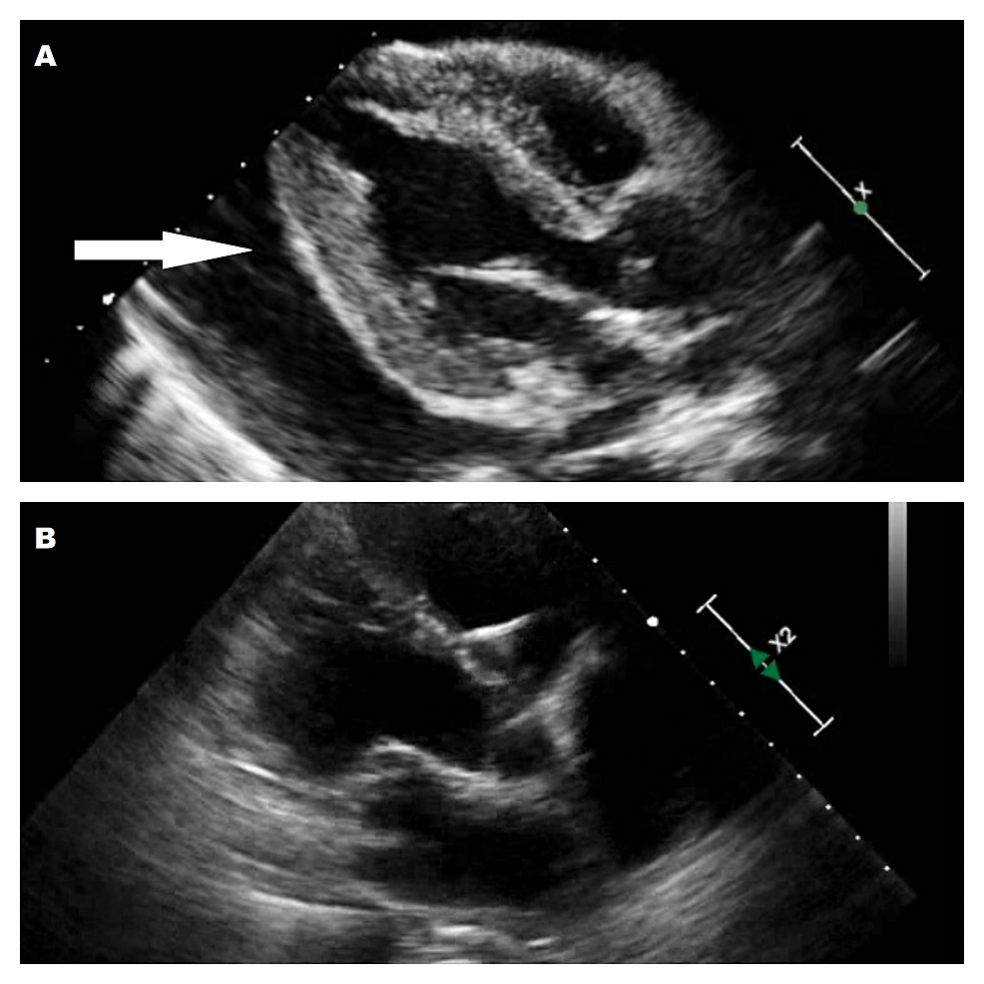
(A) Initial echocardiogram showing large pericardial effusion (white arrow) and (B) Post pericardiocentesis echocardiogram showing resolution of Pericardial effusion

The patient was started on ibuprofen and colchicine. For further evaluation of his heart, he was taken to the cardiac catheterization lab where a right heart catheterization was performed which revealed pressures consistent with tamponade physiology. During pericardiocentesis, 1200cc of serosanguinous fluid was removed which improved the patient’s symptoms remarkably (Figure 2). 

**Figure 3:**
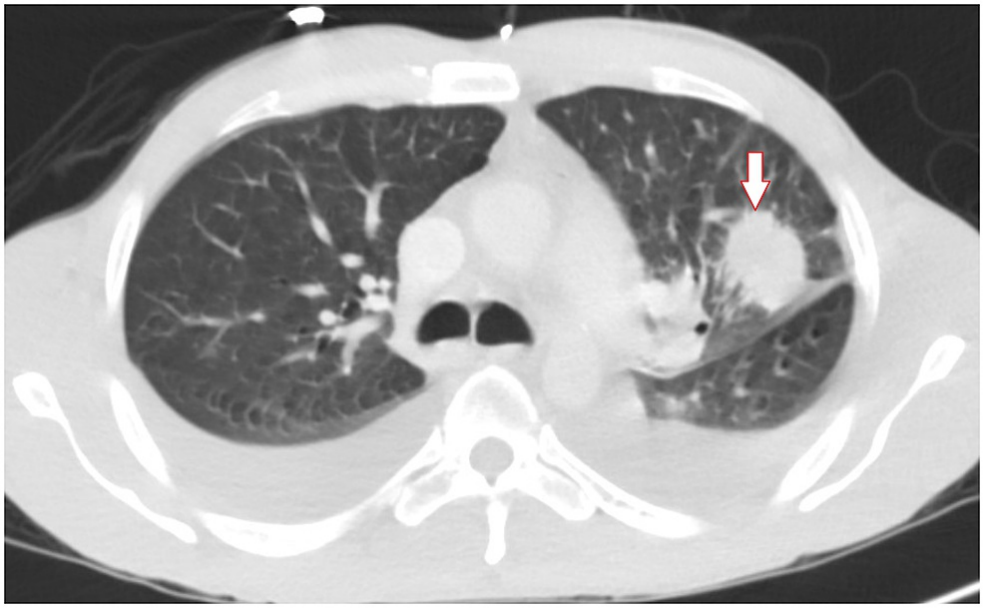
3.6 cm spiculated mass in the left upper lobe shown on CT scan of the lung (white arrow)

A sample of the pericardial fluid was sent for further evaluation. Fungal and bacterial growth was not identified in the pericardial fluid. Further blood testing was ordered. The inflammatory markers ESR was normal at 2 and CRP elevated to 10.5 mg/L. HIV test was non-reactive. Autoimmune workup including ANA, anti-DS DNA antibody, Sjogren A and B antibodies were negative. Blood tests for CEA was positive at 3.8 ng/mL (0-3ng/mL). Makers were normal for Ca 19 at 12 (<34 U/mL) and PSA at 0.8ng/mL (0.0-3.9ng/mL). A CT scan with contrast of the chest showed a spiculated mass in the left upper lobe with adjacent pleural retraction and surrounding ground glass density but no distinct enlarged mediastinal or hilar lymph nodes (Figure 3). A CT-guided left upper lobe lung mass biopsy was performed and a sample was submitted for pathological evaluation. 

The patient’s symptoms initially improved significantly with the above management but later started to experience mild to moderate chest discomfort. This chest pain prompted a follow-up ECHO which showed a recurrence of a large circumferential pericardial effusion with a reasonable likelihood of cardiac tamponade. A cardiothoracic surgery consultation was made, and the patient underwent a subxiphoid pericardial window placement. He was subsequently discharged in a stable condition to wait for the result of the pathology and immunochemical tests.

**Figure 4:**
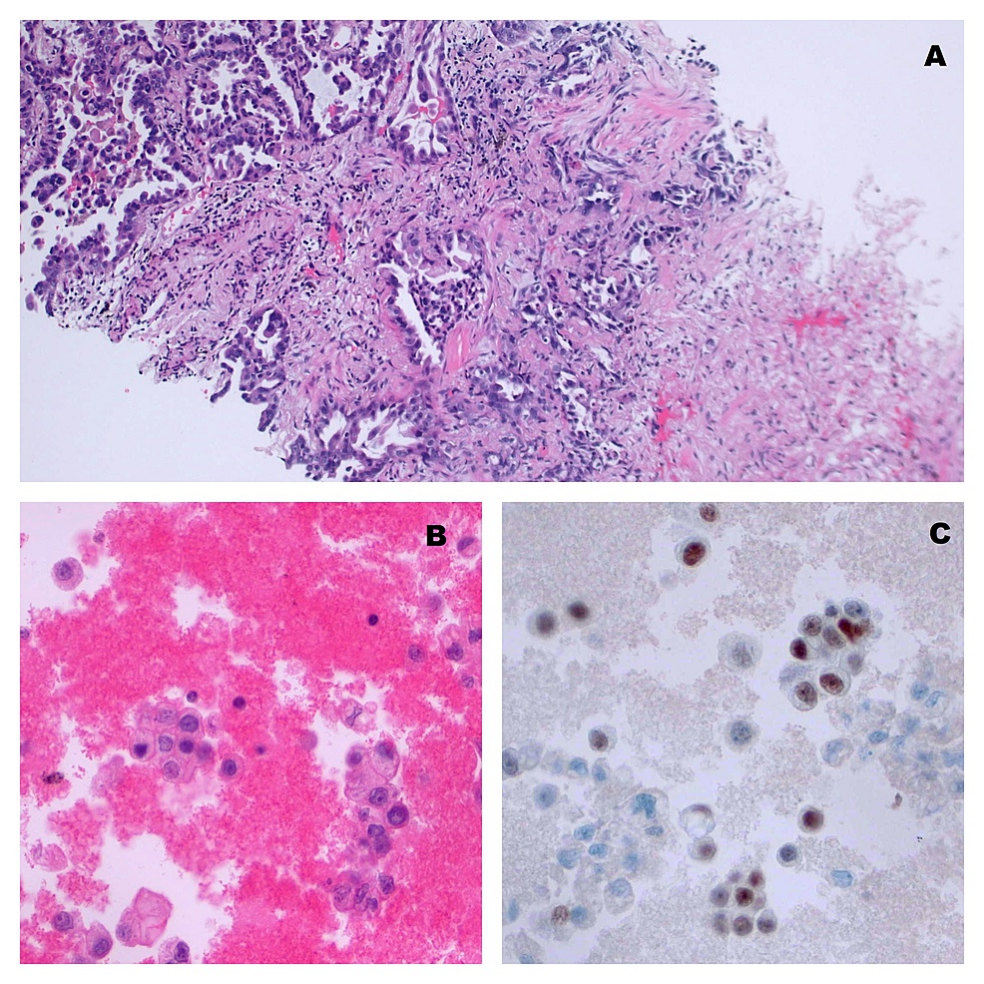
(A) Biopsy of lung showing adenocarcinoma on H & E stain, (B) pericardial fluid showing malignant cells (pap stain), and (C) pericardial fluid showing nuclear staining consistent with pulmonary adenocarcinoma (TTF1 immunostaining)

Surgical pathology from the lung mass showed atypical glandular proliferation (Figure 4). Immunohistochemical stains were positive for TTF-1, napsin A, and CK7, which is consistent with primary pulmonary adenocarcinoma. These stains were negative for CK20, WT-1, and calretinin. Molecular testing was negative for anaplastic lymphoma kinase (ALK), c-ROS oncogene 1 (ROS1), epidermal growth factor receptor (EGFR), and programmed cell death ligand 1 (PD-L1). Pathological examination of the pericardial fluid showed malignant cells and the diagnosis of stage 4 lung cancer was confirmed (Figure 4). CT scans of the abdomen and pelvis and MRI of the brain were performed with no evidence of visceral metastatic disease. The PET-CT showed disease in the lung with mediastinal adenopathy without evidence of metastatic disease other than to the pericardium. 

## Discussion

The four major histologic types of lung cancer are adenocarcinoma, squamous cell carcinoma, large cell carcinoma, and small-cell carcinoma. Adenocarcinoma accounts for about 50% of all lung cancers, making it the most common lung cancer [4]. It is also the most common lung cancer in never smokers or light smokers [5,6]. With no history of smoking and histopathologic findings consistent with adenocarcinoma, our patient was atypical in this regard. Although most lung cancers are caused by smoking, non-smoking-related lung cancer is also among the top 10 causes of cancer death in the United States. In 2021, it is estimated that non-smoking-related lung cancer will cause over 20,000 deaths [2]. Our case is a good reminder that, unlike common perception, nonsmoking-related lung cancer has a high incidence in the United States. Clinicians need to keep lung cancer in the differential diagnosis of patients presenting with pertinent signs and symptoms even if they are nonsmokers.

The diagnosis of lung cancer is mostly made following evaluation for respiratory signs and symptoms or incidental finding of a nodule or mass during routine imaging. The most common presentations of lung cancer are cough (50% to 75%), hemoptysis (25 to 50%), dyspnea (25%), chest pain (20%). Other symptoms include hemoptysis, chest pain, hoarseness, superior vena cava syndrome, and Pancoast syndrome. It can metastasize to other organs in the body including the liver, adrenal glands, bones, and brain [3]. With predominantly gastrointestinal symptoms such as nausea and epigastric pain, our patient’s presentation was very atypical in this regard. Later he was found to have a large quantity of pericardial fluid, the evaluation of which led to the identification of underlying lung cancer. This case is a good reminder that although pericardial effusion is not one of the commonly reported presentations of lung cancer, the reverse isn’t true. It has been reported that malignancy, most importantly primary lung cancer, is the cause of large symptomatic pericardial effusion in up to a fifth of cases when basic workup is non-revealing [7,8]. In our case, the initial basic evaluation didn’t reveal a potential cause for the large pericardial effusion; however, further, the workup with the CT scan of the chest revealed an underlying lung mass. This could suggest the need for investigating unrecognized malignancy in patients with large pericardial effusion with negative basic workup.

Goals for the treatment of malignant pericarditis include symptomatic relief, reversing tamponade, and prevention of recurrence. It is a common clinical practice to treat patients with malignant pericardial effusion with non-steroidal anti-inflammatory drugs and/or colchicine. Our patient also received a combination of these two medications. However, to our knowledge, there are no solid data to support this practice. Prospective studies which evaluate treatment outcomes of malignant pericardial effusion with colchicine and/or NSAIDs are needed. Patients who present with hemodynamic compromise from pericardial effusion require timely drainage of the fluid. Recurrence of pericardial effusion after pericardiocentesis is a common problem [9]. A systematic review of percutaneous interventions for malignant pericardial effusion found a recurrence rate of nearly 40% in patients who had a single pericardiocentesis [10]. Our patient complained of initially improved but gradually increasing chest pain and breathlessness and was found to have a recurrent large pericardial effusion on a repeat ECHO. To prevent the recurrence of cardiac tamponade, he underwent subxiphoid pericardial window placement. This highlights the importance of monitoring patients and obtaining a repeat ECHO as necessary following pericardiocentesis to prevent hemodynamic collapse from recurrence of large pericardial effusion.

## Conclusions

In conclusion, this case highlights that even if most cases of lung cancer are caused directly by smoking, non-smoking-related lung cancer still has a high incidence in the US, making it among the top 10 causes of cancer deaths. Even if pericardial effusion isn’t among the most common presenting symptoms of lung cancer, the reverse isn’t true. In patients who present with large pericardial effusion and have negative basic workup as in this case, a detailed evaluation for undetected underlying malignancy is important. Although colchicine and NSAIDs are commonly used in the treatment of large painful malignant pericardial effusion, there is still a lack of data to support this practice. Prospective studies looking into the outcomes of patients receiving this treatment are needed. Finally, pre-discharge screening echocardiography in patients with new or recurring cardiorespiratory symptoms following initial pericardiocentesis could be important because recurrent large pericardial effusion is a common and potentially fatal complication of malignant pericardial effusion.
